# Supplementary figures and images for: Exploration into Galectin-3 Driven Endocytosis and Lattices
Source: Biomolecules. 2024 Sep 18;14(9):1169. doi: 10.3390/biom14091169 (PMC11430376; doi:10.3390/biom14091169)

Supplemental Figure S1

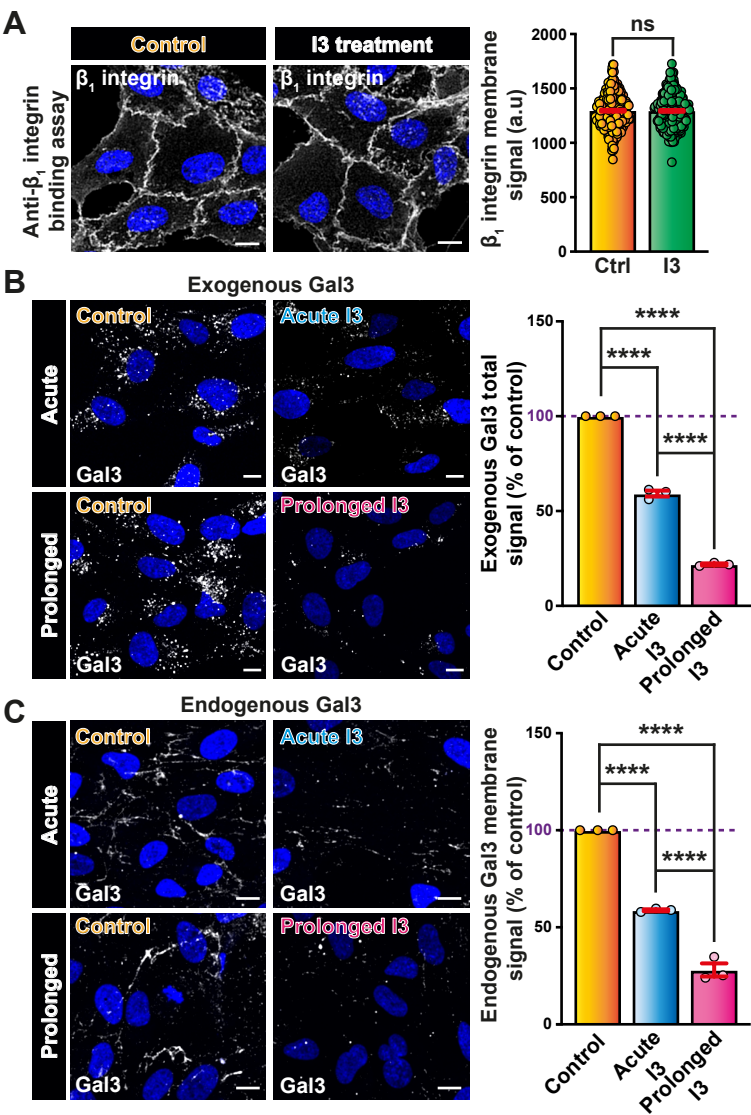

Supplement: Supplementary file 1 [file biomolecules-14-01169-s001.zip › Fig.S1-2024-09-16.pdf]

Supplemental Figure S2

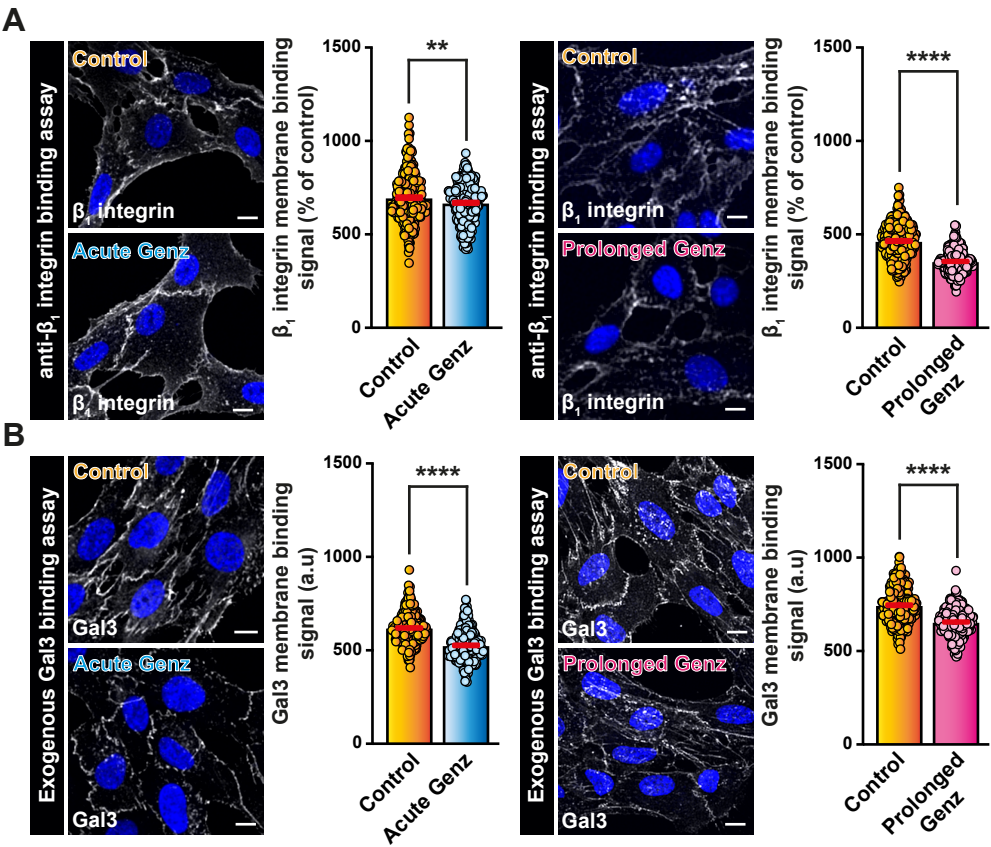

Supplement: Supplementary file 1 [file biomolecules-14-01169-s001.zip › Fig.S2-2024-09-16.pdf]
